# Sexual behaviours and their associated factors among young people in the Dodowa Health and Demographic Surveillance Site (DHDSS) in Ghana

**DOI:** 10.4314/gmj.v56i3s.6

**Published:** 2022-09

**Authors:** Mary P Agyekum, Enoch O Agyekum, Alexander Adjei, Kwabena Asare, David E Akpakli, Sabina Asiamah, Irene Tsey, Georgina Amankwah, Alfred K Manyeh, John E O Williams, David A Ross

**Affiliations:** 1 Dodowa Health Research Centre/Ghana Health Service, Dodowa, Ghana; 2 World Bank, Ghana Office; 3 School of Nursing and Public Health, University of KwaZulu-Natal, South Africa; 4 School of Public Health, University of Ghana, Legon, Accra, Ghana; 5 Institute of Health Research, the University of Health and Allied Sciences, Ho, Ghana; 6 Stellenbosch University, South Africa/ London School of Hygiene & Tropical Medicine, United Kingdom

**Keywords:** young people, sexual behaviour, condom use, pregnancy, socio-economic status

## Abstract

**Objective:**

This paper describes sexual behaviours and their associated factors among young people.

**Design:**

The study design is cross-sectional.

**Setting:**

Dodowa Health and Demographic Surveillance Site (DHDSS) in Ghana's Shai-Osudoku and Ningo Prampram districts.

**Participants:**

Young people aged 10 to 24 years, median age 17 years.

**Outcome measures:**

Self-reported to have ever had sex, non-use of a condom at last sex, and ever been pregnant or gotten someone pregnant.

**Results:**

Of the 1689 young people; 42% reported having ever had sex, not using a condom at last sexual activity (64%), and ever been pregnant or gotten someone pregnant (41%). The proportion of non-use of condoms at last sex was high across all age groups but was highest (93%) in a small proportion of 10 to 14-year-olds who have ever had sex. Higher proportions of females than males; were reported to have ever had sex (46%), not using a condom at their last sex (66%) and ever been pregnant or getting someone pregnant (56%). Age group (20 to 24), females, primary or junior high school, living alone and lower household socio-economic status were risk factors associated with all three outcome measures.

**Conclusion:**

Risky sexual behaviour is high among young people in the Dodowa HDSS. Therefore, interventions that promote safer sexual practices and help young people make timely decisions on their sexual and reproductive health care needs are required.

**Funding:**

No funding was obtained for this paper

## Introduction

The World Health Organization (WHO) defines young people as 10 to 24-year-olds, which comprises adolescents aged 10 to 19 years and young adults 20 to 24 years.^1^, ^2^ Adolescents and young adults account for a third of the population in sub-Saharan Africa (SSA).^3^ The transition from childhood to adulthood involves behaviour and lifestyle patterns that affect young people's health.^4^ This period of growth is a critical stage in life as it is characterised by risky sexual behaviours such as early sex initiation, unprotected sex, having multiple sexual partners and non-use of condoms.

These risky sexual behaviours are the predominant transmission routes for sexually transmitted infections (STIs) and often lead to unwanted pregnancies and associated unsafe abortions.^5^, ^6^ In developing countries, adolescents have special unmet sexual and reproductive health needs such as inadequate knowledge and access to comprehensive sexual and reproductive services.^7^ As a result, many are exposed to inaccurate or incomplete information. 8, 9 Adolescents who rely on inaccurate information are exposed to high-risk sexual activities, resulting in pregnancies, STIs, and unsafe abortions.

In addition, pregnant girls often drop out of school and are unlikely to be enrolled again, and many often do not have the social and economic means to raise their children.^5^, ^10^

In Ghana, young people form about one-third of the Ghanaian population. According to the Ghana Demographic and Health Survey, most unmarried young people are involved in high-risk sexual behaviours such as early sexual intercourse and the non-use of condoms during sexual intercourse.^11^ Alarmingly, from the survey, the pregnancy rate among females 15 to 19 years had increased to 76 per 1000 women in 2014 compared to 66 in 2008. As expected, there is a high knowledge of contraceptives among young people in Ghana, with male condoms widely known but yet has low utilisation among sexually active young people.^11^,^12^ Several sexual risk behaviour studies have been conducted among young people in Ghana.^11^–^16^ Factors such as gender, educational level, household socio-economic status, urban settlers, strong adult influence, and substance usage are associated with risky sexual behaviours.

Most of these studies were conducted in areas lacking Health and Demographic Surveillance Systems (HDSS). HDSS collects and updates population indicators, including demographics, pregnancy, births, deaths, migration, marriage and household socio-economic status.^17^ With these data available, it is easy to understand and relate certain health outcomes to the population within the HDSS and develop targeted interventions to mitigate their health risks. For example, young people (10 to 24 years) in the Dodowa HDSS form about 33% of the population, and out of the 1982 births recorded in 2014, about 33% were mothers aged 15 to 24 (2014 Dodowa HDSS report, unpublished). Therefore, it is important to understand the sexual behaviours among these young people. Although the data was collected in 2014, the study's findings are still relevant as there are limited studies on adolescents' sexual behaviours that combine data from health demographic surveillance sites and cross-sectional surveys. Similarly, limited studies explore adolescent sexual behaviours together with their socio-economic background and age strata as done in this study. Additionally, limited interventions have been implemented between 2014 and to date that seeks to improve adolescent sexual and reproduction health in the Dodowa HDSS; therefore, the study's findings are still relevant to the current context.

This paper describes sexual behaviours and their associated factors among young people aged 10 to 24 in the Dodowa HDSS. Understanding young people's sexual behaviours and their associated factors could direct researchers and educators to develop targeted and effective interventions to improve sexual and reproductive health behaviours among young people.

## Methods

### Study design and setting

The study was a cross-sectional survey of the Ghana site of the INDEPTH Healthy Transitions to Adulthood Study (IHTAS). The study was conducted in 2014 in the DHDSS, located within Ghana's Ningo-Prampram and Shai-Osudoku districts. The host institution for the study was the Dodowa Health Research Centre. The DHDSS has 115,754 persons under surveillance, with over 23,647 households scattered in 380 largely rural communities. 18 A detailed description of the study area can be found elsewhere.^17^

### Study population and sample size

The study population for the IHTAS involved young people aged 10 to 24 years resident in the DHDSS, who were randomly selected with population proportional to size within three age/sex strata (10–14, 15–19, 20–24 years). A sample of 1689 was estimated to be sufficient to estimate a true prevalence of 50% of contraceptive use within a 10 to 100% range in Open epi.

### Data collection tool and procedure

Data were collected electronically through a face-to-face interview with a structured questionnaire. The electronic questionnaire was designed within Open Data Kit (ODK) Collect and installed on Samsung Galaxy tablets for data collection. Data collected included demographics, young people's nutrition, hygiene, sexual, and health-seeking behaviours. This analysis uses data on the demographics and sexual behaviour module of the IHTAS study.

Data collectors were trained to administer questions using a Samsung galaxy tablet. The questionnaire for the study was pre-tested in another district outside the study area. Data collectors then visited pre-selected households and study subjects and administered questions in the local language (Dangme). Participants were assured that their responses were confidential. Before starting an interview, written informed consent and assent were administered in a language that the respondent or guardian understood. An interview took an average of 40–50 minutes and was held in an environment where the respondent could express themselves confidentially.

### Study variables

Three outcome variables were used to describe sexual behaviours among young people. These self-reported behaviours were ever had sex, non-use of a condom at last sex, and ever been pregnant or had gotten someone pregnant. The study's outcome measures were self-reported and could not be verified.

Explanatory variables used in this paper were age group (categorised into 10 to 14, 15 to 19 and 20 to 24 years), sex, education level, occupational status, type of guardian and sexual behaviour factors. Sexual behaviours factors were age at first sex, knowledge of where to get condoms, the number of sexual partners and use of a condom at first sex. Associations between sexual behaviour outcomes and alcohol usage were also assessed.

Household socio-economic status as measured by the DHDSS was also explored as an explanatory variable. Household socio-economic status was coded as either “High” or “Low” based on household sanitation, source of drinking water, housing structure, electricity and assets including a motor vehicle, bicycle and television through principal component analysis 19, 20

### Data Analysis

The data were analysed using STATA statistical software version 15.^19^. A descriptive analysis was conducted to describe the study's explanatory variables by each of the three outcomes.

Univariable and multivariable logistic regression models were fitted to identify the association of explanatory variables with each of the three study outcomes. First, uni-variable logistic regression models were fitted to explore the independent association of each exploratory variable with the corresponding outcomes. Afterwards, the logistic regression models with multivariable adjustments were fitted using all the explanatory variables to determine factors associated with each outcome. A p-value < 0.05 was considered statistically significant in a logistic regression model. Finally, results from the model were presented as tables reporting unadjusted and adjusted odds ratios with 95% confidence intervals and p-values.

### Ethical considerations

The study obtained ethical approval from the Dodowa Health Research Centre Institutional Review Board (IRB NO DHRC/260214).

## Results

### Demographics of study participants

Table 1 shows the demographic distribution of the study participants by the three outcome variables. The study had roughly equal numbers of females (51.5%) and males. Out of the 1689 participants, 42% of young people self-reported having ever had sex, 63.6% of those who reported having sex did not use condoms during their last sexual intercourse, and 41% reported being or getting someone pregnant. The proportion of young people who reported to have ever had sex or who reported having been pregnant or gotten someone pregnant increased substantially with age. The proportion of young people aged 20 to 24 years who reported having sex was high (83.3%), and a half (50.1%) of them reported being pregnant or getting someone pregnant. Higher proportions of females reported having ever had sex (46.1%), not using a condom at the last time of sex (66.1%) and ever been pregnant (56.4%). The use of condoms during first sex was less reported among sexually active 10–14-year olds as 93% of 10–14-year olds who have ever had sex did not use a condom.

**Table 1:** Description of the study's explanatory variables by the study outcomes

Factor	Level	Total N (%)	Ever had sex n (%)	Not using a condom at last sex n (%)	Ever been pregnant or gotten someone pregnant n (%)
**Total**		1689 (100)	709/1689(41.98)	451/709 (63.61)	291/709 (41.04)
**Gender**	Female	869 (51.45)	401(46.14)	265 (66.08)	226 (56.36)
	Male	820 (48.55)	308 (37.56)	186 (60.39)	65 (21.10)
**Age group**	10 - 14	580 (34.34)	27 (4.66)	25 (92.59)	2 (7.41)
	15–19	570 (33.75)	233 (40.88)	150 (64.38)	64 (27.47)
	20 -24	539 (31.91)	449 (83.30)	276 (61.47)	225 (50.11)
**Education level**	Primary	681(40.32)	148 (21.73)	118 (79.73)	81 (54.73)
	JSS/JHS	671 (39.73)	349 (52.01)	227 (65.04)	153 (43.84)
	SHS and above	337 (19.95)	212 (62.91)	106 (50.00)	57 (26.89)
**Occupational status**	Unemployed	583 (34.52)	289 (49.57)	187 (64.71)	137 (47.40)
	Employed	357 (21.14)	290 (81.23)	190 (65.52)	142 (48.97)
	Student	749 (44.35)	130 (17.36)	74 (56.92)	12 (9.23)
**Household socio-economic** **status**	High	699 (41.39)	275 (39.34)	146 (53.09)	100 (36.36)
	Low	990 (58.61)	434 (43.84)	305 (70.28)	191 (44.01)
**Guardian**	Single parent	617 (36.53)	255 (41.33)	168 (65.88)	96 (37.65)
	Both parent	612 (36.23)	170 (27.78)	100 (58.82)	44 (25.88)
	Other relatives	293 (17.35)	120 (40.96)	63 (52.50)	36 (30.00)
	Live alone/with a partner	167 (9.89)	164 (98.20)	120 (73.17)	115 (70.12)
**Heard of STI**	Yes	1316 (77.92)	660 (50.15)	413 (62.58)	267 (40.45)
	No	373 (22.08)	49 (13.14)	38 (77.55)	24 (48.98)
**Know a place to get a** **condom**	Yes	980 (58.02)	598 (61.02)	355 (59.36)	233 (38.96)
	No	709 (41.98)	111 (15.66)	96 (86.49)	58 (52.25)
**Ability to get a condom**	Yes	704 (41.74)	476 (67.52)	292 (61.34)	183 (38.45)
	No	931 (55.12)	226 (24.27)	153 (67.70)	104 (46.02)
**Age at 1st sex**	Below 16 years	203 (28.63)		153 (75.37)	67 (33.00)
	16 years and above	506 (71.37)		298 (58.89)	224 (44.27)
**Number of sexual partner**	1partner	316 (44.57)		207 (65.51)	90 (28.48)
	Multiple partners	393 (55.43)		244 (62.09)	201 (51.15)
**Used a condom at 1^st^** **sex**	Yes	252 (35.54)			78 (30.95)
	No	457 (64.46)			213 (46.61)
**Ever drunk alcohol**	Yes	634 (37.54)	408 (64.35)	251 (61.52)	174 (42.65)
	No	1055 (62.46)	301 (28.53)	200 (66.45)	117 (38.87)

More than half of young people in senior high school (62.9%) and junior high school (52.0%) reported ever having sex. Out of a small proportion (21.7%) of young people in primary school who have had sex, 79.7% reported not using a condom at last sex, and 54.7% reported ever being pregnant or making someone pregnant.

About a third (58.6%) of young people belonged to lower socio-economic status households. In these lower socio-economic status households, 43.8% reported ever having sex, 70.3% self-reported not using a condom at last sex, and 44.0% reported ever being pregnant or having gotten someone pregnant. On the other hand, a higher proportion of young people who lived alone reported ever having sex (98.2%), not using a condom at the last sex (73.2%), and having ever been pregnant or had gotten someone pregnant (70.1%). From the study, 42% of young people reported not knowing where to get a condom, and more than half (55.1%) reported their inability to get a condom if needed. A little above a quarter (28.6%) of young people reported having their first sexual debut before 16 years, and more than half (55.4%) of young people reported ever having multiple sex partners. The study also reported that 64.3% of the young people who have ever had sex also reported to have drunk alcohol ever.

### Factors associated with ever had sex

The associations between the risk factors and the reported sexual behaviour (ever had sex) are shown in Table 2.

**Table 2:** Factors associated with reporting ever having sex

Factors	Level	Ever had sex			
		Unadjusted Odds Ratio (95% CI)	P-value	Adjusted Odds Ratio (95% CI)	P-value
**Age group**	10 - 14	0.01 (0.01,0.02)	<0.001	0.04 (0.02, 0.07)	<0.001
	15 - 19	0.14 (0.10,0.18)	<0.001	0.27 (1.50, 2.69)	<0.001
	20 -24	1		1	
**Gender**	Female	1.42 (1.17, 1.73)	<0.001	2.01 (1.50, 2.69)	<0.001
	Male	1		1	
**Education level**	Primary	0.16 (0.12, 0.22)	<0.001	1.48 (0.91, 2.69)	0.115
	JSS/JHS	0.64 (0.49, 0.84)	0.001	1.69 (1.16, 2.47)	0.007
	SHS and above	1		1	
**Occupational status**	Unemployed	0.23 (0.17, 0.31)	<0.001	0.63 (0.42, 0.94)	0.023
	Employed	1		1	
	Student	0.05 (0.04, 0.07)	<0.001	0.32 (0.21, 0.48)	<0.001
**Guardian**	Both parent	1	<0.001	1	1
	Single parent	1.83 (1.44, 2.33)	<0.001	1.35 (0.97, 1.88)	0.077
	Other relatives	1.80 (1.35, 2,42)	<0.001	1.35 (0.91, 1.99)	0.131
	Live alone	142.13(44.75,451.36)		23.55(7.09,78.21)	<0.001
**Knowledge of place to get** **condom**	Yes	8.43 (6.64, 10,72)	<0.001	2.72 (1.91, 3.89)	<0.001
	No	1		1	
**Heard of STI**	Yes	6.65 (4.83, 9.15)	<0.001	1.53 (0.95, 2.47)	0.081
	No	1		1	
**Household socio-economic** **status**	Low	1.20 (0.99, 1.47)	0.065	1.76 (1.29, 2.38)	<0.001
	High	1		1	
**Ever drunk alcohol**	Yes	4.52 (3.66, 5.58)	<0.001	2.25 (1.68, 3.02)	<0.001
	No	1		1	

After adjusting for other variables, the older age group (20 to 24) and females had higher odds of reporting having sex. Young people in junior high school and those from a lower socio-economic status household had higher odds 69% and 76% respectively to report having ever had sex compared to their corresponding reference. The odds of reporting ever had sex were higher in young people who lived alone than young people who lived with both parents (AOR=23.55, 95% CI:7.03,78.21, p-value <0.001). The odds of reporting ever had sex was two times higher in young people who had ever drunk alcohol than their colleagues who had never drunk alcohol (AOR=2.30, 95% CI:1.68,3.02, p-value <0.001).

### Factors associated with the non-use of a condom at last sex

Table 3 reports the associations between risk factors and the non-use of a condom at the last sex.

**Table 3:** Factors associated with reporting not using a condom at last sex

Factors	Level	Non-condom use			
		Unadjusted Odds Ratio (95% CI)	P-value	Adjusted Odds Ratio (95% CI)	P-value
**Age group**	10 - 14	7.84 (1.83,33.49)	0.005	3.18 (0.66, 15.41)	0.151
	15 - 19	1.13 (0.82,1.57)	0.457	0.88 (0.57, 1.34)	0.542
	20 -24	1		1	
**Gender**	Female	1.28 (0.94, 1.74)	0.118	1.10 (0.78, 1.56)	0.582
	Male	1		1	
**Education level**	Primary	3.93 (2.43, 6.37)	<0.001	1.96 (1.12, 3.42)	0.018
	JSS/JHS	1.86 (1.31, 2.63)	<0.001	1.29 (0.87, 1.89)	0.200
	SHS and above	1		1	
**Occupational status**	Unemployed	0.96 (0.69, 1.36)	0.838	0.95 (0.65, 1.40)	0.816
	Employed	1		1	
	Student	0.70 (0.46, 1.06)	0.093	0.61 (0.36, 1.02)	0.058
**Guardian**	Both parent	1		1	
	Single parent	1.35 (0.91, 2.02)	0.140	1.27 (0.82, 1.96)	0.282
	Other relatives	0.77 (0.48, 1.24)	0.285	0.70 (0.42, 1.67)	0.170
	Live alone	1.91 (1.20, 3.03)	0.006	1.86 (1.11, 3.13)	0.019
**Knowledge of place to get a** **condom**	Yes	0.23 (0.13, 0.40)	<0.001	0.33 (0.18, 0.60)	<0.001
	No	1		1	
**Heard of STI**	Yes	0.48 (0.24, 0.96)	0.039	0.93 (0.43, 2.01)	0.854
	No	1		1	
**Household socio-economic** **status**	Low	2.09 (1.53, 2.86)	<0.001	1.71 (1.23, 2.41)	0.002
	High	1		1	
**Ever drank alcohol**	Yes	0.81 (0.59, 1.10)	0.178	0.88 (0.62, 1.25)	0.485
	No	1		1	
**Age at 1st sex**	Below16 years	2.14 (1.48, 3.08)	<0.001	1.86 (1.20, 2.90)	0.006
	16 years +	1		1	
**Number of sexual partners**	1 partner	1	0.347	1	0.305
	Multiple partners	0.86 (0.63, 1.17)		0.83 (0.58, 1.19)	

From the adjusted model, young people in primary school had 96% higher odds of reporting the non-use of a condom at the last sex than young people in senior high school. The odds of reporting the non-use of a condom among young people who lived alone was 86% higher than those who lived with both parents. Young people from lower socio-economic status households had 71% higher odds of reporting the non-use of a condom than young people from higher socio-economic status households. There were 86% higher odds of reporting non-use of a condom at last sex in young people who had sex before age 16 than young people who had their first sex after age 16 (AOR=1.86, 95% CI:1.20,2.89, p-value =0.006).

### Factors associated with ever been pregnant or getting someone pregnant

Table 4 presents the association of risk factors and reporting ever been pregnant or getting someone pregnant.

**Table 4:** Factors associated with reporting ever being pregnant or having gotten someone pregnant

Factors	Level	Ever been pregnant or gotten someone pregnant			
		Unadjusted Odds Ratio (95% CI)	P-value	Adjusted Odds Ratio (95% CI)	P-value
**Age group**	10 - 14	0.00 (0.00,0.00)	<0.001	0.10 (0.02, 0.61)	0.013
	15 - 19	0.18 (0.13,0.24	<0.001	0.43 (0.26, 0.71)	0.001
	20 -24	1		1	
**Gender**	Female	4.08 (3.04, 5.48)	<0.001	8.30 (5.22, 13.20)	<0.001
	Male	1		1	
**Education level**	Primary	0.66 (0.46, 0.96)	0.028	2.86 (1.51, 5.40)	0.001
	JSS/JHS	1.45 (1.04, 2.03)	0.030	1.75 (1.10, 2.80)	0.019
	SHS and above	1		1	
**Occupational status**	Unemployed	0.47 (0.35, 0.62)	<0.001	1.07 (0.68, 1.68)	0.758
	Employed	1		1	
	Student	0.02 (0.01, 0.05)	<0.001	0.23 (0.10, 0.49)	<0.001
**Guardian**	Both parent	1	<0.001	1	
	Single parent	2.38 (1.63, 3.46)	0.012	1.34 (0.80, 2.24)	0.266
	Other relatives	1.81 (1.34, 2.88)	<0.001	1.24 (0.66, 2.31)	0.503
	Live alone	28.55 (18.23, 44.72)		5.91 (3.20, 10.92)	<0.001
**Knowledge of place to get** **a condom**	Yes	3.50 (2.58, 4.76)	<0.001	0.56 (0.30, 1.04)	0.065
	No	1		1	
**Heard of STI**	Yes	3.70 (2.40, 5.72)	<0.001	0.61 (0.27, 1.39)	0.238
	No	1		1	
**Household socio-economic** **status**	Low	1.43 (1.10, 1.86)	0.008	1.33 (0.88, 2.01)	0.176
	High	1		1	
**Ever drank alcohol**	Yes	3.03 (2.34, 3.93)	<0.001	1.29 (0.85, 1.94)	0.226
	No	1		1	
**Age at 1st sex**	Below 16 years	0.62 (0.44, 0.87)	0.006	0.82 (0.49, 1.36)	0.437
	16 years and above	1		1	
**Number of sexual partners**	1 partner	1	<0.001	1	
	Multiple partners	2.63 (1.92, 3.60)		2.53 (1.65, 3.86)	<0.001
**Use condom at 1st sex**	Yes	1	<0.001	1	0.001
	No	1.93 (1.41, 2.69)		1.99 (1.31, 3.03)	

After adjusting for other variables in the adjusted model, a higher odds of ever been pregnant was reported in females than in males who reported ever getting a female pregnant (AOR = 8.30, 95% CI:5.22,13.20 p-value < 0.001).

The odds of reporting to be ever pregnant or gotten someone pregnant was highest among young people in primary school followed by those in junior high school compared to young people in senior high school (AOR = 2.86, 95% CI:1.51,5.40 p-value = 0.001).

Again, young people who live alone had higher odds of reporting ever being pregnant or getting someone pregnant than those who lived with both parents (AOR = 5.91 95% CI:3.20,10.93 p-value < 0.001). Lastly, young people with multiple sexual partners and young people who reported not using a condom at their first sex reported higher odds of ever been pregnant or getting someone pregnant.

## Discussion

This paper describes three key self-reported sexual behaviours (ever had sex, using a condom at last sex, and reporting to have ever been pregnant or have gotten someone pregnant) and examines the factors that are associated with these sexual behaviours among young people aged 10 to 24 years in the Dodowa HDSS in Ghana. The study corroborates findings on young people's sexual behaviour in sub-Saharan Africa (SSA). The results show that the proportion of young people aged 15–19 in these rural coastal districts of Ghana who reported to have ever had sex and using a condom at last sex is similar to those reported within the general Ghanaian population and in other coastal districts in Ghana.^13^, ^21^ Whereas the age at first sex among 10 -19 years old falls within the rates reported across SSA, the rate of unprotected last sex was higher in the study population than those reported across SSA^22^. Meanwhile, national surveys and studies across SSA were limited in analysing adolescent sexual behaviours and outcomes across the age spectrum of 10–24 years.

This study thus provides further insight into risky sexual behaviours among young people in rural coastal Ghana. The results show, for instance, that although a lower proportion (5%) of younger adolescents (10–14 years) were reported to have ever had sex, the non-use of a condom at last sex was very high (93%) in this age group compared to 64% in older adolescents (15–19 years) and young adults (61%). The health and long-term economic impact of adverse sexual activity among younger adolescents could be dire compared to their older cohorts, the results of this study signal the need to initiate sexual education at preadolescent age. However, a relatively lower proportion of younger adolescents might be sexually active. Studies have found that young people are likelier not to have information on their sexual and reproductive health as most first sex encounters are unplanned. 15, 23 Such sexual education to preadolescents and younger adults should project the positive benefits associated with safe sex practices, as existing evidence shows that adolescents are more driven by their perceptions of the positive benefits associated with risky behaviours rather than the knowledge of the dangers involved in risky sexual activity.^24^

This study also confirms the findings from other studies in Ghana and SSA that engaging in sex and its consequences are often adversely felt by young females more than their male counterparts.^21^,^22^,^25^ Young females might be pressured into having sex by their male counterparts or by an adult for the intention of companionship or economic incentives.^26^,^27^ Often, they may have no control or limited knowledge to bargain for using a condom during sexual encounters, which could avert pregnancies and STIs.^26^

The lower socio-economic status of households often becomes a risk factor that predisposes young people to sex, leading to pregnancies. Young people, mostly girls from lower-income households, mostly enter into sexual relationships with the intention of getting some income for themselves and their family.^26^–^28^ In most cases, these pregnancies in young people become a socio-economic burden on the household as they depend on their parents or guardians. 29 These findings provide additional justification for livelihood improvement programs that target adolescents in poor households.

Even though condoms have been well promoted in countries like Ghana, this paper found low use of condoms among young people.^25^,^30^ Some studies have found slightly higher use of condoms across Ghana's population compared to other countries such as Malawi, Uganda, and Burkina Faso.^11^,^25^,^30^,^31^ This study points to the low use of condoms at the population level could result from a significant percentage of young people having higher unmet needs for condom access and use.

The study recommends comprehensive and targeted sex education from the preadolescent stage to reduce the likelihood of unsafe sexual behaviours among young people. The household's socio-economic status plays a significant role in the sexual behavioural choice of adolescents. Livelihood empowerment programs such as cash transfers should prioritise households with young adolescents, particularly girls, to reduce the tendencies of a cycle of poverty due to adverse impacts from risky sexual behaviours.

## Conclusion

This study identified low use of condoms and high reported cases of ever been pregnant or getting someone pregnant among young people who self-reported having sex. It also identifies young females in primary or junior high school, living alone and having lower household socio-economic status as risk factors for the three sexual behaviours measured in the study.
